# Differences in prey-handling behavior of earthworm-eating snakes between a specialist and a generalist

**DOI:** 10.1093/cz/zoag001

**Published:** 2026-01-08

**Authors:** Hiroaki Yanagi, Akira Mori

**Affiliations:** Department of Zoology, Graduate School of Science, Kyoto University, Sakyo, Kyoto 606-8052, Japan; Department of Zoology, Graduate School of Science, Kyoto University, Sakyo, Kyoto 606-8052, Japan

**Keywords:** dietary specialist, earthworm, *Hebius*, prey-handling behavior, *Ptyas*, snake

## Abstract

Predators are generally classified into 2 types based on feeding strategies: dietary specialists and generalists. Specialist predators exhibit prey-handling behaviors adapted to specific prey types. In snakes, earthworm-feeding has evolved independently multiple times, resulting in the presence of both specialists and generalists among earthworm-eating species. Nevertheless, studies on prey-handling behavior of earthworm-feeding snakes remain limited. We compared prey-handling behavior of 2 colubrid snakes, an earthworm-feeding specialist, the Ryukyu green snake (*Ptyas semicarinata*) and a generalist, the Japanese keelback snake (*Hebius vibakari*), by feeding them earthworms. *Ptyas semicarinata* swallowed earthworms with minimal lateral jaw movements and exhibited significantly shorter prey-handling time than *H. vibakari*. The swallowing behavior of *P. semicarinata* partially differed from typical jaw-walking and is likely an adaptation to earthworm prey. The movements of the earthworms appeared to facilitate ingestion in *P. semicarinata*. Future studies employing CT imaging and functional analyses of cranial morphology may help elucidate the mechanisms underlying this rapid swallowing behavior.

## Graphical Abstract

**Graphical Abstract:**
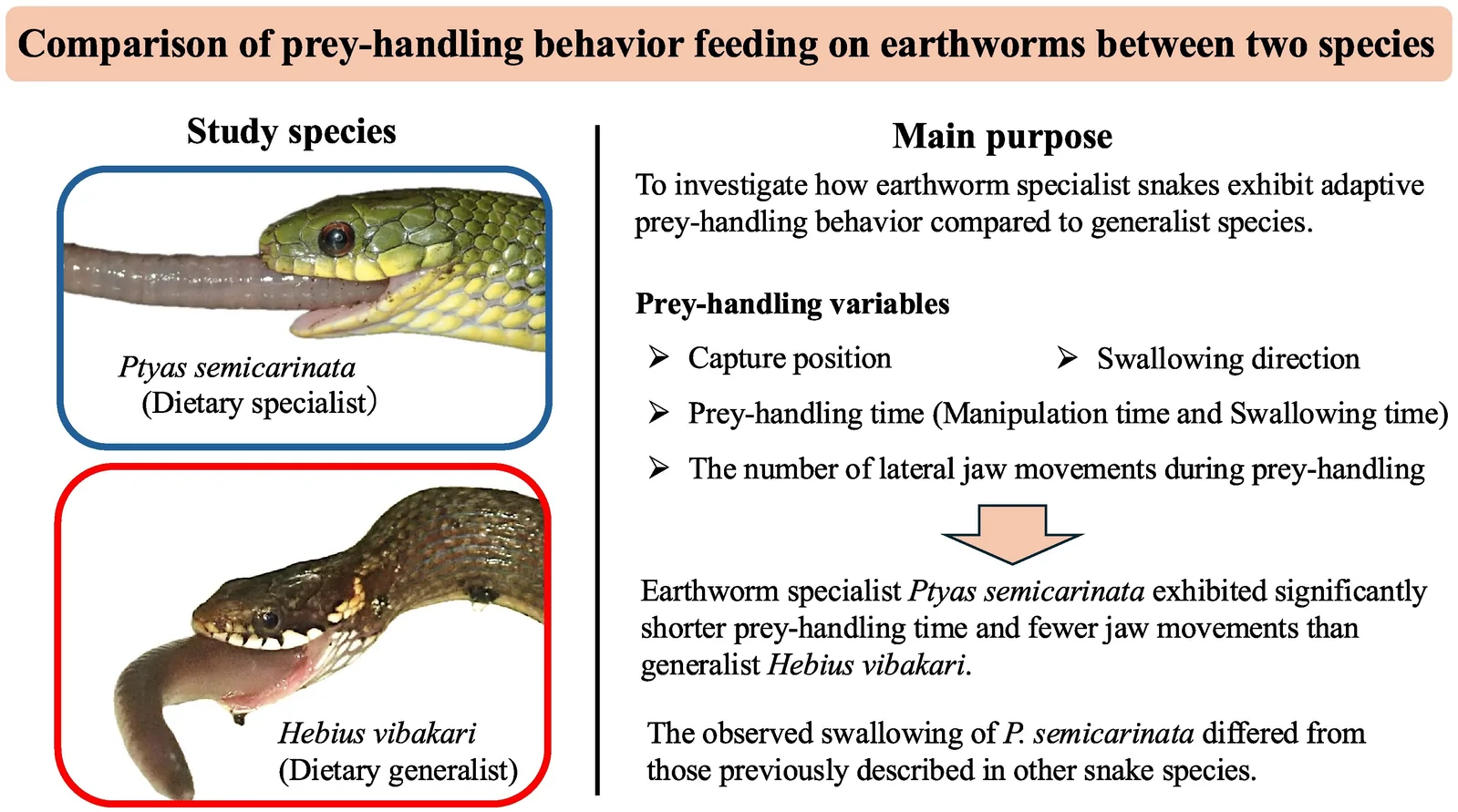

Predators require energy to sustain basic physiological functions, growth, and reproduction, and they obtain it by feeding on other animals. Thus, prey-handling behavior is one of the most important traits for their ecology and evolution (Feder and Lauder 1986). Predatory behavior not only determines individual survival and reproductive success but also shapes morphological and behavioral traits through evolutionary time (Arnold 1983; Wainwright 1994; Gripshover et al. 2023). Therefore, understanding the adaptations involved in prey capture and consumption is fundamental to comprehend animal evolution.

Predators are often classified into dietary specialists and generalists. Dietary specialization is typically associated with stable and predictable prey availability, whereas dietary generalization is favored in environments where prey availability is highly variable. These ecological differences can drive the evolution of distinct foraging behaviors and strategies (Drummond 1983; Mori 1991; Cundall and Greene 2000; Terraube et al. 2014). Specialists exhibit adaptive prey-handling behaviors for specific prey animals, while generalists exhibit flexible prey-handling behaviors suitable for consumption of various prey types (Mori and Vincent 2008; Michálek et al. 2017).

In snakes, prey-handling behavior tends to show noticeable adaptations to diet because most snakes swallow their prey whole without chewing. Feeding mechanics are particularly crucial in snakes, as they must restrain, manipulate, and swallow prey using highly kinetic skull composed of multiple loosely connected elements joined by elastic ligaments (Cundall and Greene 2000; Moon et al. 2019). Therefore, dietary specialization primarily in terms of the functional demands associated with prey capture and swallowing is expected in snakes. Furthermore, snakes exhibit remarkable dietary diversity, with different species preying on a wide variety of animals, making them excellent models for clarifying the relationship between prey-handling behavior and diet (Lillywhite 2014).

Many snakes are capable of swallowing whole prey that exceeds the diameter of their own heads. This remarkable ability has attracted considerable interest from anatomists and ethologists and has been studied extensively (Cundall and Greene 2000; Moon et al. 2019; Segall et al. 2023). Much of this research has focused on the adaptations that enable the ingestion of large prey. However, some snakes feed exclusively or primarily on slender prey animals such as fossorial lizards, eels, centipedes and earthworms. Feeding on slender prey poses distinct mechanical challenges compared with ingesting bulky prey. For example, species with large gape sizes may have difficulty in securely grasping and manipulating elongated prey animals, which can easily slip from the jaws before ingestion begins. Specialized prey-handling behaviors may have evolved to overcome these challenges, yet studies focusing on such behaviors in slender prey specialists are still limited (Burghardt and Krause 1999; Kley and Brainerd 2002; Jackson et al. 2004; Gripshover et al. 2023; Fukuda et al. 2025). Radcliffe and Chiszar (1980) showed that a sea snake species swallows an eel remarkably quickly, implying that specialized behavioral and morphological traits for quick ingestion of slender prey. Earthworms, as prey, present additional challenges: they are extremely flexible, capable of secreting mucus, and, in some species, can autotomize their tails to escape predators (Emberts et al. 2019). These features could necessitate even more specialized prey-handling strategies for successful capture and ingestion.

Here, we focus on earthworm-feeding snakes and compare prey-handling behavior between a dietary specialist and generalist. Earthworm-feeding snakes have evolved independently multiple times within the snake lineage, resulting in a diversity of species that includes both dietary specialists and generalists (O'Shea 2023; Fukuda et al. 2025). This repeated evolution of earthworm-feeding, along with the presence of both types of dietary strategies, makes them an excellent model system for examining behavioral specialization for slender prey.

In Japan, several snake species are known to feed on earthworms. Among them, we focused on 2 colubrid species with different dietary habits: the Japanese keelback snake (*Hebius vibakari*) and the Ryukyu green snake (*Ptyas semicarinata*). *Hebius vibakari* is a small common snake distributed on the Japanese mainland and is considered a dietary generalist, because it eats morphologically diverse animals such as frogs, fish, and earthworms (Mori and Moriguchi 1988; Hamanaka et al. 2014), whereas *P. semicarinata* is a medium-sized snake distributed in the Central Ryukyu Islands and is considered a dietary specialist that feeds almost exclusively on earthworms (Fukuyama et al. 2025; Yanagi, unpublished data). Based on these different dietary habits, we compared prey-handling behavior toward earthworms between the 2 species. We predicted that *P. semicarinata* exhibits prey-handling behavior that efficiently subdue and process earthworms. In addition, because earthworms have several defense responses, such as mucus and autotomy, we expected that *P. semicarinata* may exhibit prey-handling behavior to overcome these defenses. Thus, we recorded any defensive behaviors of earthworms observed during the feeding experiment.

## Materials and methods

### Study species

We collected a total of 18 individuals of adult *P. semicarinata* from Okinawa and Amami Islands, and their snout-vent length (SVL) ranged from 644 to 1,015 mm (mean ± SD = 853 ± 116 mm). Eighteen individuals of adult *Hebius vibakari* were collected from Kyoto, and their SVL ranged from 293 to 414 mm (345 ± 31 mm). They were housed individually in plastic cages containing shelters and water bowls, with paper lay on the bottom. The minimum cage size used was 410 mm × 240 mm × 140mm and 190 mm × 120 mm × 130 mm for *P. semicarinata* and *H. vibakari*, respectively.

### Laboratory experiments

The experiments were conducted in the home cages after removing the shelters and water bowls or in another larger cage after transferring the snakes to it. They were allowed to acclimate for at least 10 min before the experiment. We placed earthworms in the cage and recorded prey-handling behavior by video camera (Sony FDR-AX55 and Apple iPhone 12). All earthworms used as food were megascolecid species. We calculated relative prey size (RPS) as the ratio of the maximum body width of the earthworm (mm) to the maximum head width of the snake (mm). Because measuring body width of live earthworms was difficult due to their contraction. We measured body mass and estimated the body width of live earthworms from body mass. We first weighed earthworms while alive and measured their body width after fixation in formalin. Both variables were log-transformed and a regression line between body mass and body width was determined. Based on the regression line, which showed a highly positive correlation (*R*^2^ = 0.96, *P* < 0.001, Supplementary Figure S1), body width was estimated from body mass for each earthworm. The laboratory air temperature was 26–28 °C, and the experiments were conducted in a bright condition. Before the experiments, snakes were deprived of food at least for 3 days. In *P. semicarinata*, data were collected 3 times from 16 individuals and twice from 2 individuals, resulting in a total of 52 trials. In *H. vibakari*, data were collected twice from each of 18 individuals, resulting in 36 trials.

We analyzed the video movie and compared the following 5 variables between the 2 species. (1) Capture position: the earthworm's body part grasped by a successful strike by the snake. Three capture positions were defined: anterior part (anterior 1/3 of the body), middle part (from the anterior 1/3 to the posterior 1/3 of the body), and posterior (posterior 1/3 of the body). (2) Swallowing direction: anterior first, middle first, or posterior first. The definition of the body parts was same as capture position. Middle first ingestion indicates that a snake folded an earthworm in the region of middle and swallowed it. (3) Prey-handling time: prey-handling sequence was divided into manipulation and swallowing phases. (3-1) Manipulation time: time from successful strike of the prey to the commencement of intraoral prey transport. A successful strike was defined as the most recent strike in which the prey was not released at any point before swallowing was completed. The commencement of intraoral prey transport was defined as the time when we observed the first lateral movement of the jaw in the direction in which the prey was subsequently swallowed (i.g. when part of the earthworm's body was taken into the snake's mouth toward the cervical direction). (3-2) Swallowing time: time from the commencement of intraoral prey transport to the first tongue flick after ingestion. (3-3) Total prey-handling time: Sum of manipulation time and swallowing time. (4) The total number of lateral jaw movements during manipulation. (5) The total number of lateral jaw movements during swallowing. In general, snakes handle their prey during manipulation and swallowing by alternately moving their left and right jaws (jaw-walking) (Cundall and Greene 2000; Moon et al. 2019). We counted the number of complete cycles of alternating jaw movements (i.e., one left-right or right-left sequence was counted as one movement) during swallowing. When earthworm autotomy occurred, no variable other than capturing position was recorded. One individual of *P. semicarinata* moved out of the camera frame during swallowing, making it impossible to count the number of jaw movements, and thus, this trial was excluded from the analysis of jaw movements.

### Statistics analysis

We used generalized linear mixed models (GLMM) with gamma distribution and log link function to compare prey-handling time (manipulation time, swallowing time, and total prey-handling time, separately) between *P. semicarinata* and *H. vibakari*. In the models, we set manipulate time, swallowing time, or total prey-handling time as dependent factor, RPS, snake species, and their interaction as independent factor, and individuals ID as random factor. We used GLMM with a negative binomial distribution and log link function to compare the number of jaw movements in the manipulation and swallowing phase separately, with RPS, species, and their interactions as independent factor and individual ID as random factor. To examine whether the RPS influenced the probability of prey repositioning, we conducted a logistic regression analysis with repositioning (0 = no, 1 = yes) as a binary response variable and RPS as the explanatory variable. All statistical analyses were conducted using R ver.4.2.2 (R Core Team 2022) and the following R packages: lme4 ver. 1.1-31 (Bates et al. 2015) and glmmTMB ver. 1.1.7 (Brooks et al. 2017). The significance level was set at 0.05.

## Results

### Prey-handling behavior

During prey-handling, both snake species used only their jaws and did not use their body to subdue the earthworms, and the earthworms were swallowed alive. During the prey-handling process from the successful strike to prey passing the quadratomandibular joint, *H. vibakari* kept closing at least 1 side of the jaws and advanced on the prey by jaw-walking (Figure 1, Movie 1). In contrast, *P. semicarinata* exhibited typical jaw-walking but advanced a greater distance more than the prey per jaw-walking cycle, accompanied by lateral head movements and cervical undulatory locomotion (Figure 2, Movie 2). It appeared to rely on cervical transport rather than jaw-walking. *Ptyas semicarinata* did not firmly securing the prey with its jaws. During the peculiar intraoral prey transport, the earthworms were swallowed very quickly. It appeared as if the earthworms were moving into the snake's mouth by themselves.

**Figure 1:**
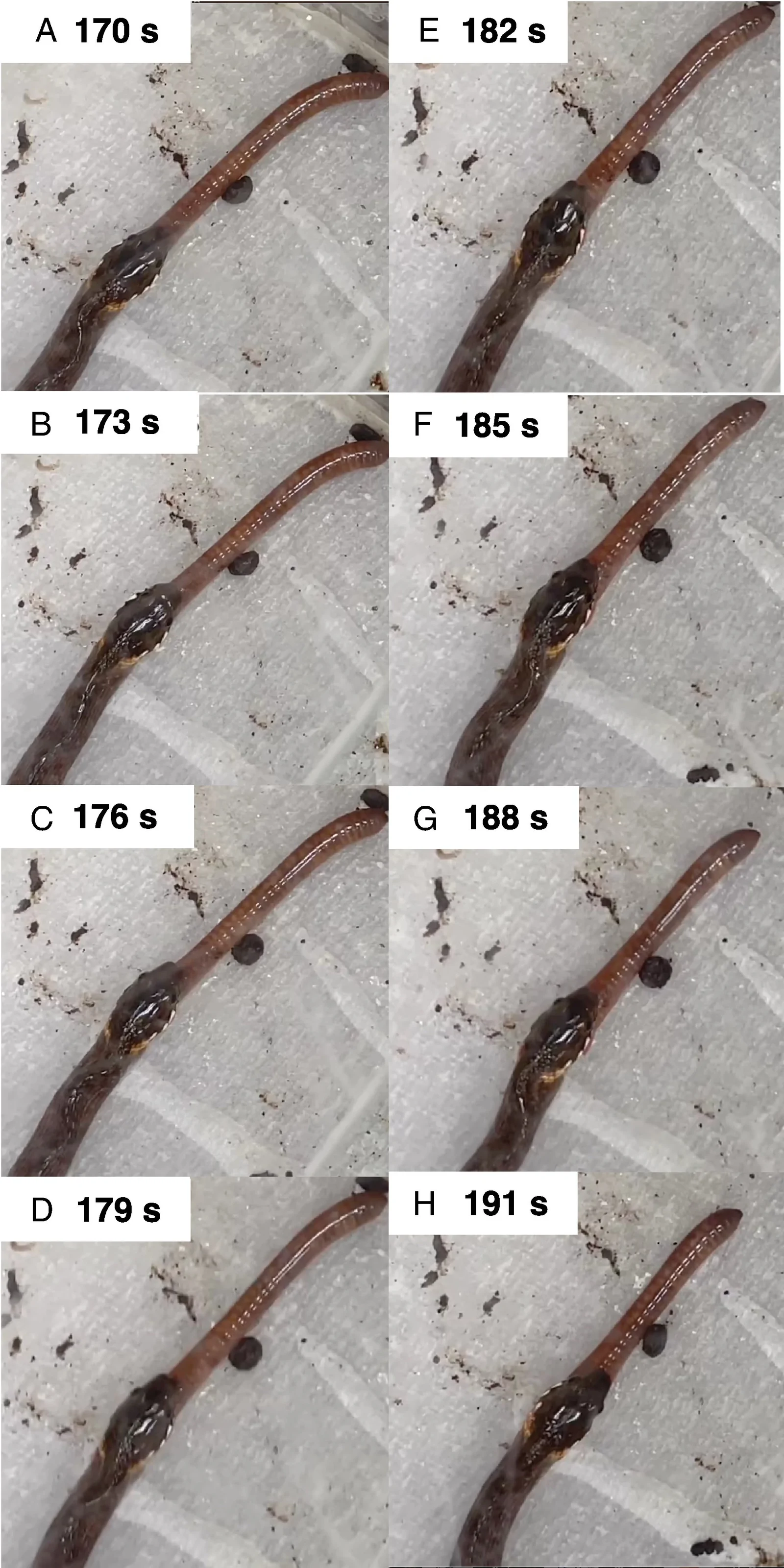
Intraoral prey transport of a *Hebius vibakari* (SVL 315 mm) swallowing an earthworm (BM 2.6 g, RPS = 0.75). Panels (A–H) show frames at 3 s intervals more than a 21 s period from 170 to 191 s after the commencement of swallowing. Six cycles of jaw-walking were observed during this interval, with the snake moving forward more than the prey for approximately 2 head lengths. The neck remained relatively straight.

**Figure 2:**
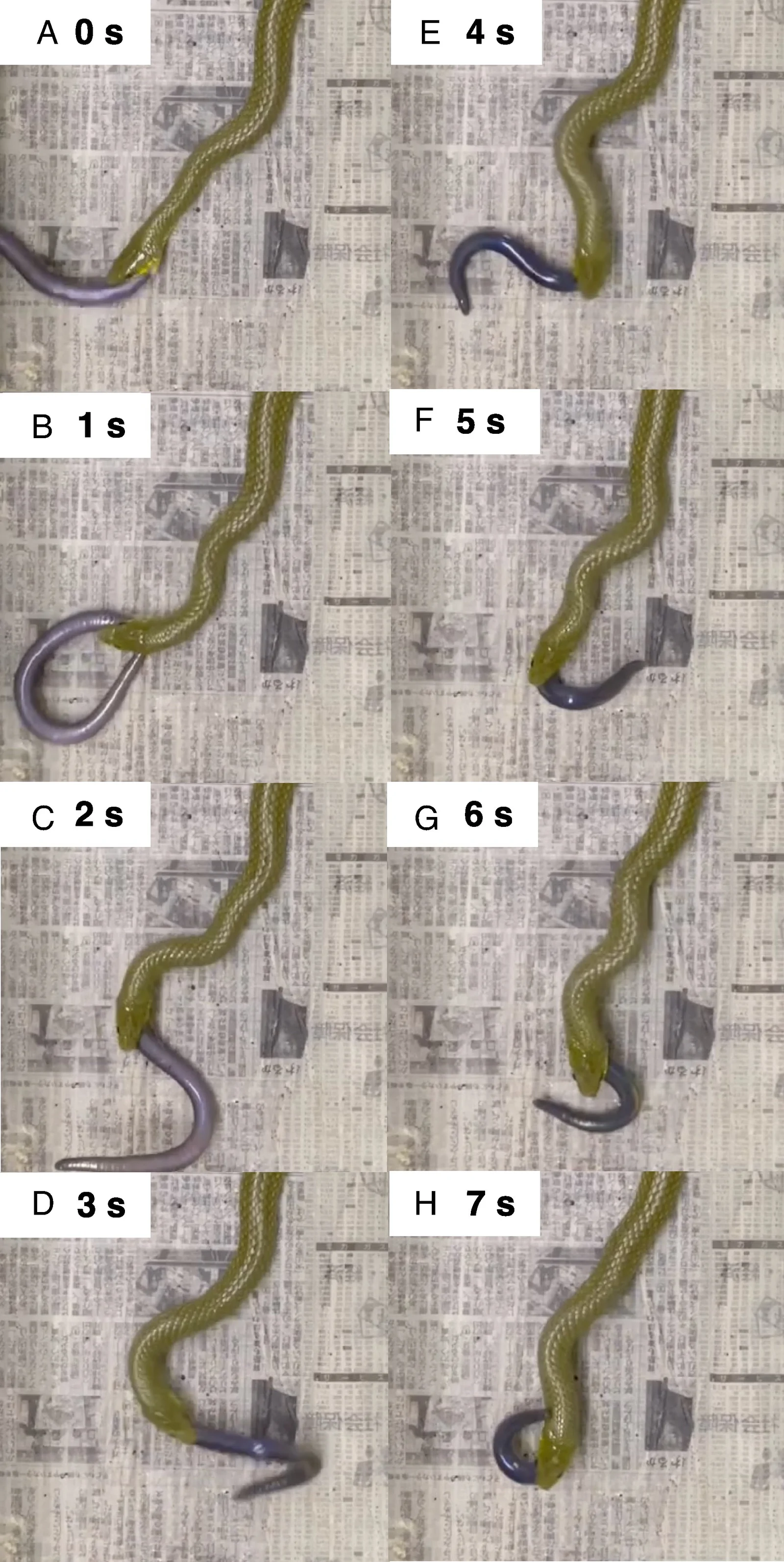
Intraoral prey transport of a *Ptyas semicarinata* (SVL 706 mm) swallowing an earthworm (BM 5.6 g, RPS = 0.87). Panels (A–H) show frames at 1 s intervals more than a 7 s period from 0 to 7 s after the commencement of swallowing. Eight cycles of jaw-walking were observed during the interval. Notably, the snake advanced more than the prey by a distance clearly exceeding that expected from a single jaw-walking cycle, moving forward more than the prey for approximately 5 head lengths. During this swallowing process, the neck exhibited undulatory movements.

*Hebius vibakari* exhibited only a single strike failure, and the snake did not attempt to release the prey after striking. In contrast, *P. semicarinata* released the prey immediately after striking on 11 cases. The behavior appeared to reflect intentional release rather than true strike failure.

### Defensive behavior of prey

Defensive behaviors of earthworms included mucus secretion, tail autotomy, and coiling around the snake head. However, both species of snakes avoided the mucus, and no earthworm prevented predation by secreting mucus Autotomy of earthworms was observed 3 times in each snake species when snakes bit the posterior body of earthworms. After autotomy, *P. semicarinata* was observed eating only the detached tail on 2 occasions, leaving the body uneaten. In contrast, *H. vibakari* consumed both the tail and body in all autotomy cases. Additionally, although coiling might have delayed swallowing, it was insufficient to deter predation.

### Capture position and swallowing direction

Both snake species tended to bite the anterior part of the prey [64% (23/36) in *H. vibakari* and 50% (26/52) in *P. semicarinata*], although middle and posterior bites were also observed (Table 1). Posterior bites tended to led to the earthworm's autotomy [75% (3/4) in *H. vibakari* and 18% (2/11) in *P. semicarinata*]. *Hebius vibakari* tended to swallow the prey from the initial bite position. When biting the middle part of the earthworm, *H. vibakari* folded the prey and swallowed it from there in all cases. In contrast, when *P. semicarinata* bit the prey in the middle, the snake either folded the prey and swallowed it from there or repositioned the prey and swallowed it from the posterior. The mean RPS was 0.56 (SD = 0.048, *n* = 5) when *P. semicarinata* folded the prey for swallowing without repositioning after biting the middle part. In cases where the prey was repositioned prior to swallowing, the mean RPS was 0.68 (SD = 0.16, *n* = 9). Logistic regression analysis showed that RPS was not a significant predictor of repositioning of prey (*z* = 0.82, *P* = 0.41).

**Table 1:** Swallowing direction in relation to bite position in *Ptyas semicarinata* and *Hebius vibakari*.

Bite	*Hebius vibakari*			*Ptyas semicarinata*		
	Anterior	Middle	Posterior	Anterior	Middle	Posterior
**Swallow**						
Anterior	22	0	0	24	1	0
Middle	1	9	0	0	5	0
Posterior	0	0	1	2	8	9
(Autotomy)	0	0	3	0	1	2

### Prey-handling time and jaw movements

Summary statistics (means ± SD) for total prey-handling time, manipulation time, swallowing time, and the number of lateral jaw movements during each phase for each species are provided in Supplementary Table S1.

In total prey-handling time, RPS had a significant positive effect, whereas species did not have a significant effect. However, the interaction between RPS and species was significant, indicating that the effect of RPS on prey-handling time differed between species (RPS: *z* = 4.33, *P* < 0.001; species: *z* = 0.78, *P* = 0.43; RPS × species: *z* = −2.11, *P* = 0.035). The effect of RPS on prey-handling time was weaker in *P. semicarinata* than in *H. vibakari* (Figure 3). In manipulation time, none of the independent variables had a significant effect (RPS: *z* = 1.87, *P* = 0.06; species: *z* = − 0.75, *P* = 0.45; RPS × species: *z* = 0.58, *P* = 0.56). In contrast, in swallowing time, as in total prey-handling time, only RPS and the interaction between RPS and species showed significant effects (RPS: *z* = 4.04, *P* < 0.001; species: *z* = 1.36, *P* = 0.17; RPS × species: *z* = −2.79, *P* < 0.01).

**Figure 3:**
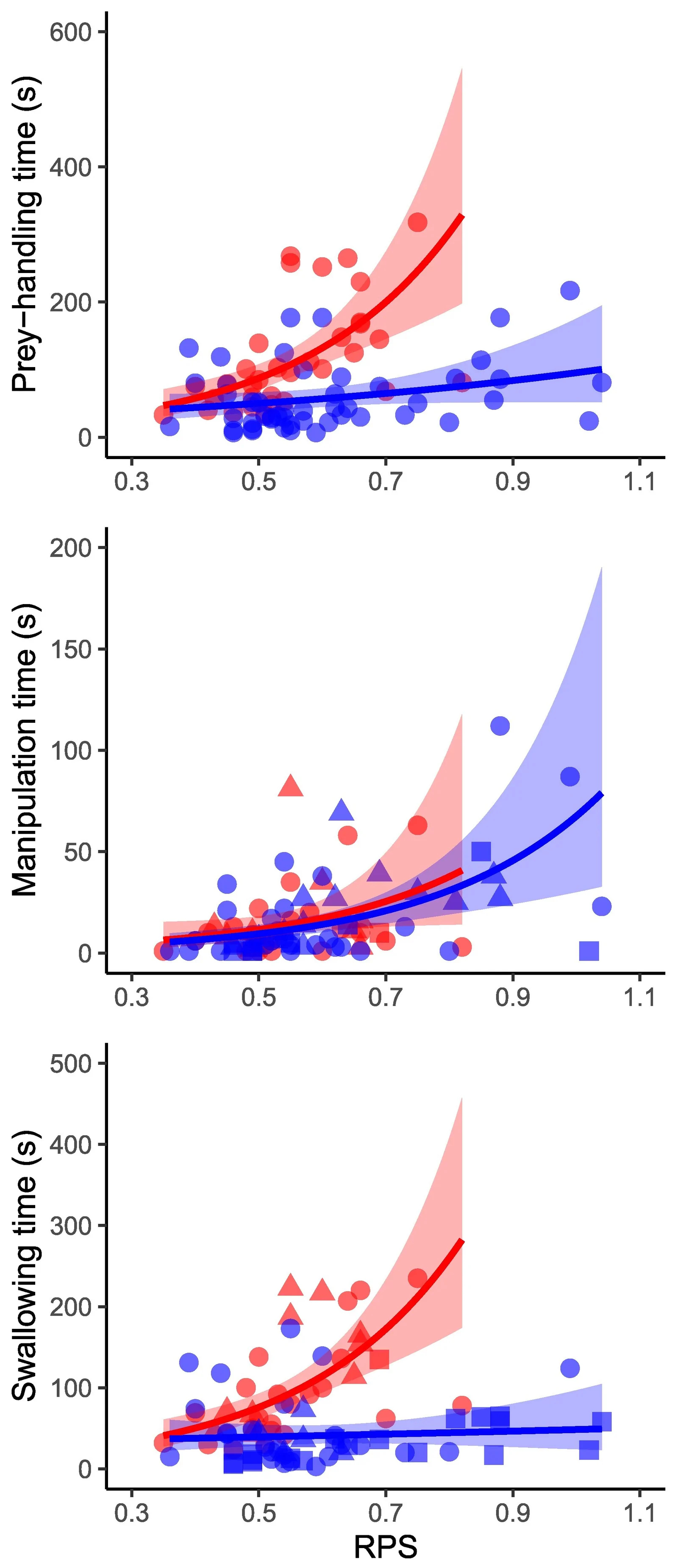
(Above) Relationships between RPS and total prey-handling time in *Ptyas semicarinata* and *Hebius vibakari*. (Middle) Relationships between RPS and manipulation time. The shape of plots indicates bite position: circle: anterior, triangle: middle, square: posterior. (Below) Relationships between RPS and swallowing time. The shape of plots indicates swallowing direction: circle: anterior, triangle: middle, square: posterior. In all panels, blue line and plots indicate *P. semicarinata*, and red line and plots indicate *H. vibakari*.

For the total number of lateral jaw movements, only RPS had a significant effect in both the manipulation and swallowing phases; neither species nor the RPS × species interaction showed significant effects (Manipulation: RPS: *z* = 2.20, *P* = 0.028; species: *z* = 1.17, *P* = 0.24; RPS × species: *z* = −1.52, *P* = 0.13; Swallowing: RPS: *z* = 2.03, *P* = 0.042; species: *z* = 0.41, *P* = 0.68; RPS × species: *z* = −1.31, *P* = 0.19: Figure 4). However, in 17 cases of *P. semicarinata* swallowing was completed in 7 or fewer jaw cycles, which was never observed. The mean swallowing time in those 17 cases was 22.1 s (SD = 14.5).

**Figure 4:**
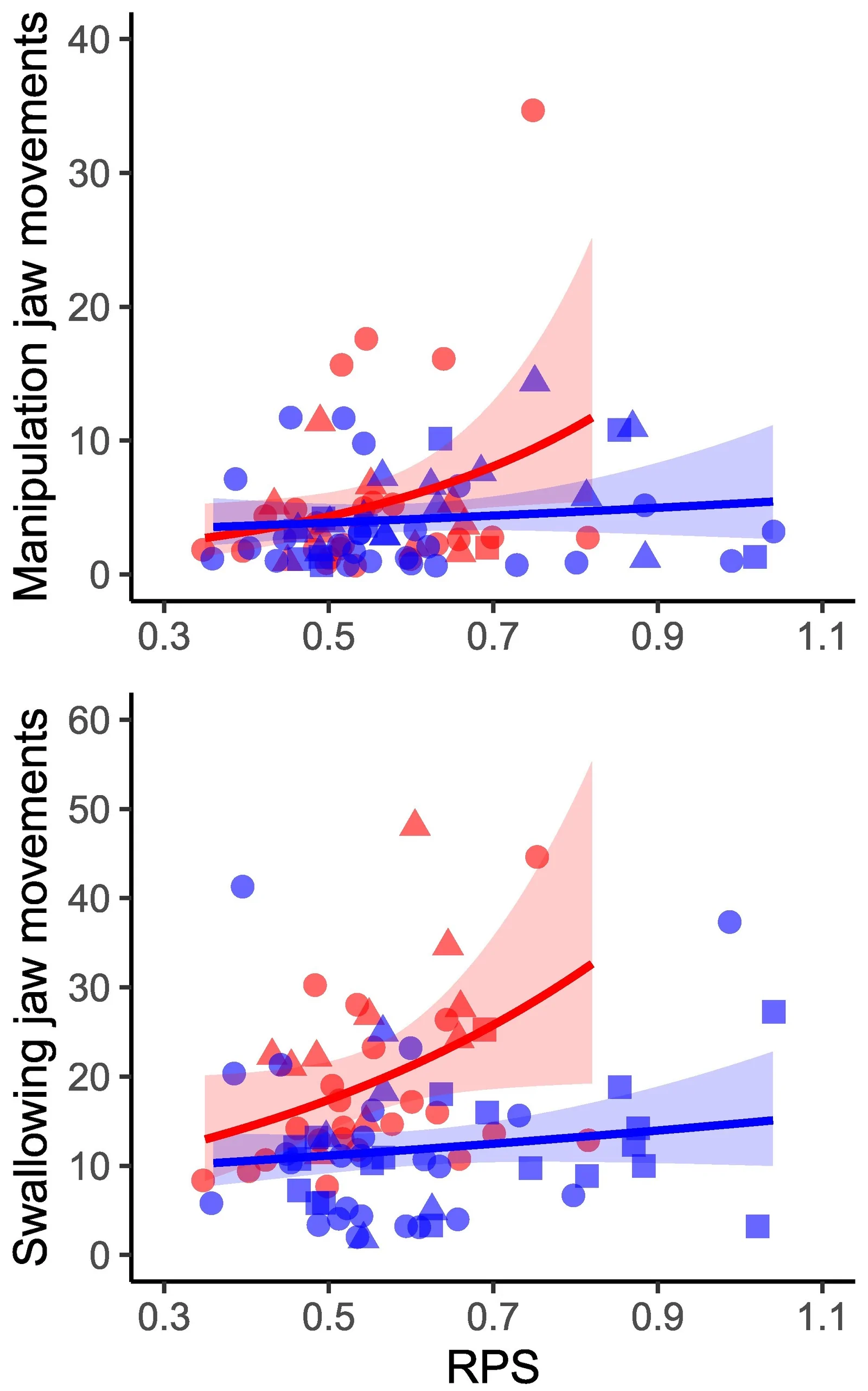
(Above) Relationship between RPS and the number of lateral jaw movements during manipulation phase in *Ptyas semicarinata* and *Hebius vibakari*. The shape of plots indicates bite position: circle: anterior, triangle: middle, square: posterior. (Below) RPS and the number of lateral jaw movements during swallowing phase. The shape of plots indicates swallowing direction: circle: anterior, triangle: middle, square: posterior. In all panels, blue line and plots indicate *P. semicarinata*, and red line and plots indicate *H. vibakari*.

## Discussion

We predicted that prey-handling behavior of the specialist, *P. semicarinata*, would reduce the likelihood of eliciting defensive responses of earthworms. However, defensive behaviors were observed in both the specialist and generalist species in a similar frequency. Thus, the ability of *P. semicarinata* to suppress prey defenses may be limited. Nonetheless, the observed defensive responses appeared to have little effect on both snake species. Given that earthworms are preyed upon by a wide range of terrestrial predators, their defensive behaviors such as mucus secretion and tail autotomy may have evolved mainly to counter nonsnake predators, potentially rendering these defenses less effective against snakes.

The high frequency of anterior biting observed in both snake species is likely attributable to the active movements of the earthworm's anterior region, which the snakes presumably use as a cue to locate their prey. Several species of snakes reposition prey to start swallowing from a preferred end, rather than from the initial bite position (e.g., Rodríguez-Robles 2002; Jackson et al 2004; Mori 2006; Tumlison and Roberts 2018). In our study, *H. vibakari* typically began to swallow the earthworms from the initial bite position regardless of the position, suggesting little selectivity in swallowing direction. This result is consistent with a recent finding that *H. vibakari* tends to swallow earthworms from the initial bite location without selective repositioning (Yoden and Mori 2025). On the other hand, when *P. semicarinata* bit the middle part of the earthworms, repositioning of the prey occurred in more than half of the cases. Although the difference in RPS between cases with reposition and fold was not statistically significant, the mean RPS tended to be higher when repositioning occurred. The lack of a significant difference may have been due to the limited sample size. This finding may indicate that larger prey items are more difficult to fold and thus require repositioning for effective ingestion. A similar behavioral pattern was suggested in *Rhabdophis chiwen*, supporting the notion that prey size influences prey-handling strategies in earthworm-eating species (Fukuda et al. 2025). Moreover, Yoden and Mori (2025) pointed out that opportunistically foraging snakes are expected to prioritize minimizing the risk of prey escape more than reducing prey-handling time. Therefore, if repositioning increases the risk of prey escape, snakes are presumed to initiate swallowing from the initial bite position rather than attempting to reposition the prey. However, in earthworm-feeding snakes, the encounter rate with prey is likely to be higher than in vertebrate-feeding snakes, because earthworms are detritivores that occupy a lower trophic position in terrestrial ecosystems and generally occur at high densities in soil environments. It suggests that minimizing prey-handling time may be prioritized more than minimizing escape risk in earthworm-eating snakes. In addition, *H. vibakari*, a dietary generalist, preys on vertebrates such as frogs and fish, which are considered more nutritionally valuable than earthworms (Reguera et al. 2011). Therefore, its prey-handling behavior may be more strongly adapted to ensuring the secure capture of such high-value prey, rather than to efficiently processing earthworms. This may explain its relatively less specialized handling of earthworms observed in our study.

Total prey-handling time and swallowing time showed significant effects of RPS as well as a significant interaction between RPS and species. In contrast, manipulation time was significantly affected only by RPS. These results suggest that the interspecific interaction effect on total prey-handling time mainly arises from the swallowing phase. *Ptyas semicarinata* was able to swallow large prey within a relatively short time, whereas its manipulation time was not reduced. Although this species often released the prey once after striking, the behavior did not appear to shorten the manipulation phase. The adaptive significance of the behavior remains to be clarified in future studies.

As in many other snakes, both species exhibited an increase in the number of jaw movements with increasing prey size (Pough and Groves 1983). However, in several feeding cases of *P. semicarinata* exhibited extremely few jaw movements during swallowing, and these cases would be associated with notably short swallowing times. This finding suggests that *P. semicarinata* possesses a unique behavior that allows rapid swallowing without jaw-walking. Nevertheless, such behavior was not observed consistently across all trials. Further detailed observations are needed to clarify the conditions under which this rapid swallowing occurs.

Although the detailed mechanism of swallowing in *P. semicarinata* remains unclear, we hypothesize that *P. semicarinata* may exploit the movement of the earthworm during ingestion. Many earthworm species dig underground tunnels and move within them by pressing against the tunnel walls with bristles on their body surface (Quillin 1999). Thus, it is possible that, after being partially ingested, the earthworm may actively move into the snake's esophagus under its own locomotion. Furthermore, the undulating movement of the snake's cervical region may assist the prey's progression into the esophagus. In most snakes, the initial phase of swallowing involves jaw-walking, characterized by the alternate advancement of the left and right jaws, and in the later phase, movements of the neck and body are used to transport the prey further into the stomach (Kley and Brainerd 2002). In the case of earthworms, their elongated bodies allow parts of the prey to enter the esophagus even during the early phase of swallowing, potentially enabling neck movements to assist ingestion from the beginning. The swallowing behavior observed in *P. semicarinata*, that is, transporting the prey using lateral head and neck movements without securely holding it with the jaws, presumably stimulates the earthworm's movement and facilitates rapid ingestion. The earthworms were swallowed particularly rapidly when *P. semicarinata* opened its mouth and laterally moved its head and neck without performing jaw-walking. In contrast, in *H. vibakari* one side of the jaw consistently held the prey throughout most of the swallowing process, likely preventing the earthworm from advancing voluntarily. Future studies using X-ray videography to analyze jaw and cervical vertebra movements will provide further insights into the unique swallowing mechanism of *P. semicarinata*.

Earthworm species differ in their habitat preferences, with some species dwelling under leaf litter and others burrowing tunnels underground (Ishizuka and Minakoshi 2014). In the present study, the experiments were conducted in an open environment under captive conditions. In the wild, snakes may forage in narrow spaces such as under stones or litter and within tunnels when preying on earthworms. Future field studies on diet composition, including species-level identification of consumed earthworms, and foraging behavior of *P. semicarinata* will provide a more detailed understanding of its foraging ecology and help clarify the adaptive significance of prey-handling time reduction. Previous studies have suggested that a reduction in prey-handling time contributes to decrease predation risk and energy expenditure and increase foraging efficiency (Cundall and Irish 2022; Fukuda et al. 2025). Earthworms are known to have low nutritional values, and most earthworm-eating snakes tend to be small-sized species (Reguera et al. 2011). In contrast, *P. semicarinata* is a medium-sized species, with SVL of 750–1,000 mm, and is likely the largest known earthworm-specialist (Matsui and Mori 2021). Therefore, we presume that *P. semicarinata* requires frequent foraging, which may have driven the evolutionary reduction in prey-handling time. Comparing feeding behavior, ecology, and trophic morphological features of *P. semicarinata* with those of other small earthworm-specialist snakes (e.g., species of Xenodermidae, Calamariinae, and Uropeltidae) would provide further insights into the evolutionary adaptations associated with earthworm-feeding. Accumulation of such comparative data from different lineages may help evaluate whether convergent evolutionary solutions have arisen in adaptions to earthworm-feeding among snakes.

## Supplementary Material

zoag001_Supplementary_Data

## Artificial intelligence (AI)-assisted technologies

We used artificial intelligence (AI)–assisted tools (ChatGPT-4o) only for minor language editing and for troubleshooting error messages in R code. All data analyses, interpretations, and scientific conclusions were performed by the authors.
