# Supplementary material for: Differences in prey-handling behavior of earthworm-eating snakes between a specialist and a generalist
Source: Curr Zool. 2026 Jan 8;72(5):723–30. doi: 10.1093/cz/zoag001 (PMC13690598; doi:10.1093/cz/zoag001)
Supplement: Supplementary file 1 [file zoag001_supplementary_data.zip › FigS1.pdf]

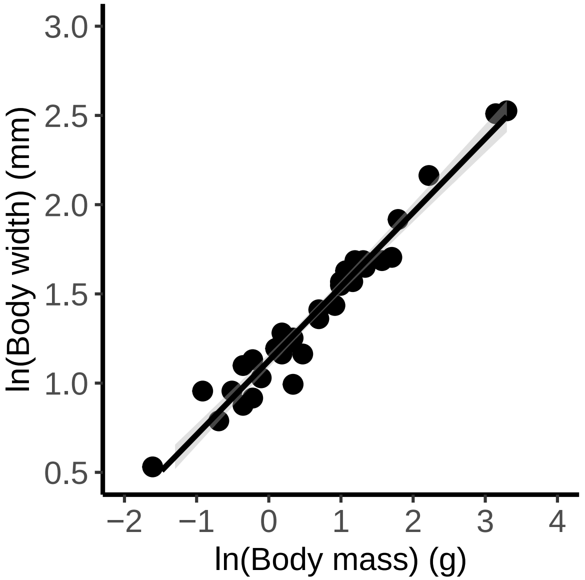
3.0
2.5
2.0
1.5
1.0
0.5
ln(Body width) (mm)
−2
−1
0
1
2
3
4
ln(Body mass) (g)
