# Supplementary material for: Differences in prey-handling behavior of earthworm-eating snakes between a specialist and a generalist
Source: Curr Zool. 2026 Jan 8;72(5):723–30. doi: 10.1093/cz/zoag001 (PMC13690598; doi:10.1093/cz/zoag001)
Supplement: Supplementary file 1 [file zoag001_supplementary_data.zip › Supplementary_Legends.docx]

Table S1. The mean of each prey-handling variable in *Ptyas semicarinata* and *Hebius vibakari*. Values are presented as mean ± SD**.**

Figure S1. Relationship between body mass and maximum body width of earthworms. Measurements were natural log-transformed.

Alt text: Scatter plot showing the relationship between maximum body width and body mass of earthworms, indicating a strong positive correlation.
